# Prevalence and predictors of suicidality among adults initiating office-based buprenorphine

**DOI:** 10.1186/s13722-023-00393-y

**Published:** 2023-06-01

**Authors:** Michelle R. Lent, Karen L. Dugosh, Emily Hurstak, Hannah R. Callahan, Kimberly Mazur, The Greater Philadelphia Opioid Use Disorder Research Group, S Festa, D DeJoseph, J DeJoseph, R DeBates, T Bulan, A Harnett, A van Riper, C Millard, H Wooten, E Loscalzo, M Leonard, O Oojewoye, D Festinger, C Lavin, E Garber, A Reichert, J McKay, K Lynch, K Kampan, C Petro

**Affiliations:** 1 School of Professional and Applied Psychology, Philadelphia College of Osteopathic Medicine, 4190 City Avenue, Rowland Hall, Philadelphia, PA 19131 USA; 2 Research and Evaluation Group, Public Health Management Corporation, 1500 Market Street, Philadelphia, PA 19102 USA; 3 Boston University School of Medicine, 801 Massachusetts Avenue, 2nd floor, Boston, MA 02118 USA; 4 AtlantiCare HealthPlex, 1401 Atlantic Avenue, Atlantic City, NJ 08401 USA

**Keywords:** Suicide, Opioid use disorder, Risk factors

## Abstract

**Background:**

Individuals who have substance use disorders may have an elevated risk of suicidality. This study sought to examine the prevalence of, and identify factors associated with, suicidality in adults with opioid use disorder (OUD) initiating office-based buprenorphine treatment.

**Methods:**

Individuals were eligible to participate if they had OUD and had initiated treatment in the past month. Participants (*n* = 244) completed a semi-structured interview using the Addiction Severity Index—Lite.

**Results:**

At baseline, 37.70% of participants reported significant thoughts of suicide over their lifetime and 27.46% reported suicidal attempts over their lifetime. Logistic regression analyses were used to identify predictors of lifetime suicidal thoughts and attempts. A history of physical abuse (OR = 4.31, *p* < .001), having chronic pain-related conditions (OR = 3.28, *p* < .001), a history of depression (OR = 3.30, *p* = .001) or anxiety (OR = 7.47, *p* = .001), and Latino/a/x ethnicity (OR = 2.66, *p* = .01) were associated with an increased risk of lifetime suicidal thoughts. A history of sexual abuse (OR = 2.89, *p* = .01), Latino/a/x ethnicity (OR = 4.01, *p* < .001), a history of depression (OR = 4.03, *p* = .001) or anxiety (OR = 15.65, *p* = .007) and having a chronic pain-related condition (OR = 2.43, *p* = .01), were associated with an increased risk of lifetime suicide attempts.

**Conclusions:**

Results demonstrate the high prevalence of suicidal thoughts and attempts among patients initiating buprenorphine. Findings may help to better identify at-risk patients and to inform screening, prevention, and mental health treatment efforts.

*Trial registration*: ClinicalTrials.gov, NCT04650386 (registered 12 December 2020, https://clinicaltrials.gov/ct2/show/NCT04650386) and NCT04257214 (registered 5 February 2020, https://clinicaltrials.gov/ct2/show/NCT04257214).

## Prevalence and predictors of suicidality among adults initiating office-based buprenorphine treatment

According to recent estimates provided by the National Institute of Mental Health [1], approximately 4.9% of U.S. adults in the general population experienced thoughts of suicide (i.e., thinking about, considering, or planning suicide) and 0.5% engaged in a suicide attempt (i.e., a non-fatal, self-directed, potentially injurious behavior with intent to die) in the past year. Patients with opioid use disorder (OUD) may be particularly vulnerable to suicidality, as individuals reporting at least weekly opioid misuse are more likely to both plan for suicide and to carry out attempts compared to individuals not misusing opioids [2].

A number of studies have demonstrated a heightened risk of suicide mortality among individuals who have substance use disorders [3, 4]. Findings from a case–control study [4] found users of illicit drugs to be more than five times as likely to die by suicide over a 13-year study period (adjusted OR = 5.3). A 2017 evaluation of suicide mortality in more than four million Veterans Health Administration patients found women with OUD to be particularly vulnerable to suicide mortality, though men with OUD were also at risk [3]. In most situations, it is very difficult to distinguish between accidental and intentional overdoses and overdose deaths. For this reason, suicidal behaviors may be underreported in this population. Capturing the true prevalence of suicide mortality in patients with OUD is challenging given the limitations of overdose surveillance and in many cases, the inability to ascertain intent [5].

Medication for opioid use disorder (MOUD) is currently considered the gold standard for the treatment of OUD and buprenorphine in particular is associated with improvements in suicide risk [6, 7]. In the general population, chronic pain, psychiatric co-morbidity including depression, schizophrenia, substance misuse, and personality disorders, a history of abuse or incarceration, and male gender are associated with elevated suicide risk, while Latino/a/x or Hispanic ethnicity is associated with lower rates of completed suicide [8–13]. Little is known, however, about whether certain patients taking MOUD may be at higher risk for suicidality. The present study aimed to: (1) assess the rates of lifetime suicidal ideation and attempts reported in a sample of adults with OUD taking buprenorphine; and (2) to evaluate the clinical and demographic factors associated with suicidality in individuals with OUD taking buprenorphine to inform suicide screening and prevention efforts.

## Methods

### Sample

A total of 244 adults (18 years or older) with OUD who were initiating office-based buprenorphine treatment at one of five Federally Qualified Health Centers (FQHCs) in Eastern Pennsylvania and New Jersey were enrolled in one of two randomized, controlled trials (Study 1 *n* = 159; Study 2 *n* = 85). Participating FQHCs were located in urban and suburban areas and provided comprehensive primary care services including mental health treatment. Both studies were designed to evaluate the effectiveness of providing adjunctive psychosocial treatment (i.e., cognitive-behavioral therapy and peer support services) to patients receiving buprenorphine treatment for their OUD. Because the studies targeted the same population, utilized the same inclusion criteria and research assessments, and employed similar methodologies, baseline data from the two studies were combined to form the analytic sample for the current paper. Study 1 began enrollment in July 2020 and Study 2 began in October 2020; both studies were actively recruiting at the time of this analysis. As seen in Table 1, 68% of participants identified as male, 38.5% as White, 38% as Black, and 25% as Hispanic or Latinx. At baseline, participants reported a mean age of 43.23 years (*SD* = 11.16). The most common medical conditions endorsed were chronic pain (37%), hypertension (36%), and respiratory problems (29%). On average, participants reported having 2.16 conditions (*SD* = 1.86; range = 0–9).

**Table 1:** Characteristics of Participants with Opioid Use Disorder (N = 244)

	*M*	*SD*	n	%
Age (years)	43.23	11.16		
Identified GENDER				
Male			65	67.62
Female			78	31.97
Transgender			1	< 1.00
Race				
Black			92	37.70
White			94	38.52
Multi-Race			14	5.74
American Indian			5	2.05
Hawaii/Pacific Islander			1	0.44
*Spanish/Puerto Rican/Latinx			38	15.57
Ethnicity				
Hispanic/Latino/a/x			62	25.41
Marital status				
Married			23	9.43
Widowed			11	4.51
Separated			19	7.79
Divorced			32	13.11
Never Married			157	64.34
Unknown			2	< 1.00
Medical Conditions (most common)*				
Chronic pain-related conditions			90	36.89
Hypertension			87	35.66
Chronic respiratory/breathing problems			70	28.69
Hepatitis			56	22.95
Diabetes			29	11.89
Heart disease			21	8.61
Epilepsy or seizures			19	7.79
History of serious depression			172	70.49
History of serious anxiety			193	79.10
Days worked (past 30 days)	5.77	9.34		
Education completed (years)	11.93	2.41		
Legal history				
On parole or probation (yes/no)			61	25.00
Months incarcerated (if applicable)	52.33	83.32		

^*^participants may have reported more than one condition; participants endorsed “Other” and then filled in their identified race

Across both studies, individuals were eligible to participate if they had moderate to severe OUD based on the number of symptoms endorsed, had initiated office-based buprenorphine treatment in the past 28 days, and displayed no evidence of psychiatric instability or cognitive impairment. Potentially eligible participants were identified by FQHC providers or staff and then introduced to trained, onsite research staff. Interested individuals were screened for eligibility, informed about the study, and then guided through the informed consent process. Following randomization, participants completed the baseline assessment battery that included a number of standardized assessments including the Addiction Severity Index-Lite (ASI-Lite; 14; see description below) and provided a urine specimen to screen for recent substance use. Participants were compensated $50.00 for their time. Studies were approved by the lead institutions’ review boards.

### Transparency and openness

These trials are currently ongoing and upon completion, we will report how we determined our sample size, any data exclusions or manipulations, and all measures in the study. Data from all participants enrolled in the studies to date were examined; cases with missing data for the predictor and/or outcome variable were excluded from the corresponding logistic regression analysis. For the present analyses, no exclusions or manipulations were conducted. Data and code will be available upon study completion at https://doi.org/10.5061/dryad.stqjq2c5n.

### Measures

All data used in the present analyses were obtained from the ASI-Lite [14], a reliable and valid multidimensional assessment of current and lifetime psychosocial functioning across seven domains commonly impacted by substance use (i.e., medical, employment, psychiatric, alcohol use, drug use, legal, and family/social). Although the instrument can be used to produce summary scores for each domain, individual binary items were used in the analyses including those measuring:

- Lifetime [1] thoughts of suicide (seriously considered a plan for taking their life and [2] lifetime suicide attempts;
- Years of heroin and other illicit opioid use and years of polysubstance use (including alcohol);
- Presence of a chronic pain-related condition and other medical conditions;
- History of physical abuse (caused person physical harm) and history of sexual abuse (forced sexual advances/acts);
- History of serious depression and anxiety; and
- Demographic information including gender identity, race, ethnicity, employment, education, legal history, and age.

### Data capture and statistical approach

All study data were collected and managed using REDCap (Research Electronic Data Capture) hosted at the University of Pennsylvania [15]. Descriptive statistics were calculated to characterize the sample at study entry and to generate prevalence rates for lifetime suicidal ideation and attempts. Next, a series of bivariate correlations were calculated between each of the binary suicide-related response variables (i.e., lifetime ideation, lifetime attempts) and clinical and demographic factors that may be associated with suicidality (age, education completed [years], gender identity [male/female with additional responses added for non-binary, etc.], race [White, Black, Other], ethnicity, years of heroin use and other opioid use, lifetime history of depression or anxiety [yes/no], physical and/or sexual abuse [yes/no], presence of a chronic pain-related condition [yes/no], parole/probation [yes/no], days worked [past 30 days], and lifetime years of daily use of more than one substance [including alcohol]). Finally, a series of logistic regression analyses were performed to predict each of the suicide-related binary response variables. Predictors that reached statistical significance (i.e., *p* < . 05) for at least one of the suicide-related response variables were included in regression models.

## Results

**Fig. 1:**
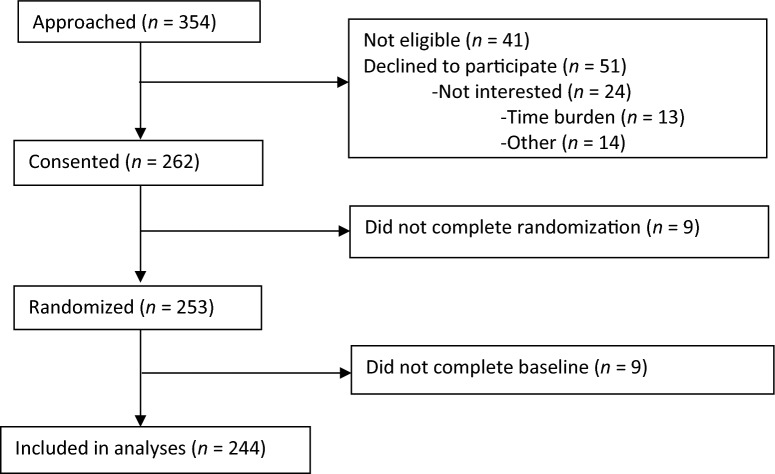
Participant Flow

At the time of this interim analysis, 354 individuals were approached about the studies, 262 consented (74%), and 244 completed baseline assessments and are included in the present analyses (68%, Fig. 1).

### Rates of suicidal thoughts and attempts

At baseline, 92 participants (37.7%) reported experiencing thoughts of suicide over their lifetime. Additionally, 67 participants (27.5%) endorsed attempts over their lifetime.

### Predictors of suicidality

All predictors were explored separately for each binary suicidality outcome. Age, gender, years of heroin use or other opioid use, days employed (past 30 days), and race were not significantly associated with lifetime suicidality outcomes (*p* > 0.05 for all). Ethnicity, years of education, lifetime depression, anxiety, physical abuse and sexual abuse, having a chronic pain-related condition, lifetime polysubstance use, and being on parole or probation significantly correlated with at least one suicidality outcome (*p* < 0.05) and were included in regression models (Table 2).

**Table 2:** Bivariate and Multivariate Predictors of Lifetime Suicidality (N = 244)

		Bivariate		Multivariate*					
		*r*	*p*	*B*	*S.E*	*OR*	*95% CI*	*Wald*	*P*
Lifetime thoughts	Gender (male vs. female)	0.17	0.008	− 0.14	0.39	0.87	0.41–1.87	0.13	0.72
	Ethnicity (non vs/ Latino/a/x)	0.15	0.02	0.98	0.38	2.66	1.26–5.62	6.57	0.01
	Education completed (years)	− 0.03	0.64	− 0.02	0.07	0.99	0.86–1.12	0.50	0.82
	Physical abuse (lifetime no vs. yes)	0.41	<0.001	1.46	0.38	4.31	2.05–9.06	14.79	<0.001
	Sexual abuse (lifetime no vs. yes)	0.31	< 0.001	0.53	0.41	1.69	0.75–3.79	1.64	0.20
	Polysubstance use (lifetime no vs. yes)	0.14	0.02	0.01	0.02	1.01	0.99–1.06	0.85	0.36
	Chronic pain (no vs. yes)	0.29	< 0.001	1.19	0.33	3.28	1.72–6.27	12.95	< 0.001
	On parole or probation (no vs. yes)	− 0.13	0.04	− 0.62	0.40	0.54	.25–1.18	2.40	0.12
	Serious depression (lifetime no vs. yes)	0.31	< 0.001	1.20	0.36	3.30	1.64–6.65	11.22	0.001
	Serious anxiety (lifetime no vs. yes)	0.33	< 0.001	2.01	0.65	7.47	2.22–25.18	10.52	0.001
Lifetime suicide attempts	Gender (male vs. female)	0.16	0.01	− 0.39	0.41	0.68	0.30–1.52	0.89	0.35
	Ethnicity (non vs. Latino/a/x)	0.19	0.003	1.39	0.40	4.01	1.84–8.71	12.24	< 0.001
	Education completed (years)	− 0.13	0.04	− 0.07	0.96	0.93	0.82–1.07	0.96	0.33
	Physical abuse (lifetime no vs. yes)	0.31	< 0.001	0.77	0.42	2.15	0.95–4.88	3.37	0.07
	Sexual abuse (lifetime no vs. yes)	0.34	< 0.001	1.06	0.43	2.89	1.24–6.77	6.00	0.01
	Polysubstance use (lifetime no vs. yes)	0.17	0.008	0.03	0.02	1.03	1.00–1.06	3.26	0.07
	Chronic pain (no vs. yes)	0.25	< 0.001	0.89	0.35	2.43	1.24–4.77	6.62	0.01
	On parole or probation (no vs. yes)	− 0.03	0.69	− 0.24	0.42	0.79	0.35–1.81	0.31	0.58
	Serious depression (lifetime no vs. yes)	0.29	< 0.001	1.34	0.44	4.03	1.72–9.45	10.27	0.001
	Serious anxiety (lifetime no vs. yes)	0.25	< 0.001	2.71	1.03	15.65	2.10–116.96	7.19	0.007

^*^Logistic regression

### Lifetime thoughts

A history of serious depression (OR = 3.30, 95% CI [1.64–6.65],* p* = 0.001) and anxiety (OR = 7.47, 95% CI [2.22–25.81], *p* = 0.001) were associated with elevated risk of lifetime suicidal thoughts. Hispanic or Latino/a/x ethnicity was associated with an elevated risk of lifetime suicidal thoughts, OR = 2.66, 95% CI [1.26–5.62], *p* = 0.01, Table 2. A lifetime history of physical abuse was significantly associated with an increased risk of lifetime history of suicidal thoughts, OR = 4.31, 95% CI [2.05–9.01], *p* < 0.001. Chronic pain-related conditions were associated with an increased risk of lifetime suicidal thoughts, OR = 3.28, 95% CI [1.71–6.27], *p* < 0.001.

### Lifetime attempts

Similarly, a history of serious depression (OR = 4.03, 95% CI [1.72–9.45)],* p* = 0.001) and anxiety (OR = 15.65, 95% CI [2.10–116.93], *p* = 0.007) were associated with elevated risk of lifetime attempts. Hispanic or Latino/a/x ethnicity was associated with greater risk of lifetime suicide attempts, OR = 4.01, 95% CI [1.84–8.71], *p* < 0.001, Table 2. Lifetime history of sexual abuse increased the risk of lifetime attempts, OR = 2.89, 95% CI [1.24–6.77], *p* = 0.01, as did having a chronic pain-related condition OR = 2.43, 95% CI [1.24–4.77], *p* = 0.01.

## Discussion

In our study, more than 37% of individuals initiating office-based opioid treatment (buprenorphine) reported experiencing suicidal thoughts over their lifetime, and 27% reported engaging in suicide attempts over their lifetime. These rates are notably higher than lifetime prevalence rates of ideation (15.6%) and attempts (5.0%) reported in the U.S. general population [16]. Given the very high prevalence of lifetime suicidality in our sample, regular screening and monitoring for suicidality may be warranted in individuals initiating MOUD given the challenges in multiple life domains that can accompany both OUD and recovery.

Several patient-level factors were related to an increased risk of suicidality. Participants who endorsed a history of physical abuse were more likely to report having suicidal thoughts over their lifetime and those with a history of sexual abuse were more likely to report a lifetime history of attempts. These findings are consistent with the body of evidence suggesting that the experience of abuse is related to suicidality [9]. Additionally, having a chronic pain-related condition (e.g., fibromyalgia, arthritis, migraines) increased the risk of lifetime suicidal thoughts as well as attempts. This is in accordance with previous findings that individuals with chronic pain are more likely to engage in suicidal behaviors [11]. Chronic pain is common among individuals with OUD, with estimates ranging from 30–60% of patients [17]. Patients with comorbid chronic pain syndromes and OUD may face particular challenges in accessing effective treatments to improve function and quality of life given the stigma impacting perceptions of OUD, as well as poor access to multimodal pain treatment approaches. Additionally, the psychiatric co-morbidities of both anxiety and depression were associated with notable elevations in suicidality risk for thoughts and attempts. Finally, identifying as Hispanic or Latino was associated with an elevated risk of endorsing lifetime suicidal thoughts and lifetime suicide attempts.

As mentioned above, all of our participants were prescribed buprenorphine for the treatment of OUD. A randomized, controlled trial conducted by Yovell and colleagues [18] found low-doses of buprenorphine to be associated with reductions in suicidal thoughts in non-substance users with severe suicidal ideation. Degenhardt et al. [19] found mortaliy risk to be reduced during methadone use but to increase during transitions off methadone; further, buprenorphine/naloxone was associated with a decreased risk of unintentional overdose. In chronic pain patients, Cameron and colleagues [20] hypothesized observed reductions in suicide risk to be associated with buprenorphine’s potential to minimize pain-induced aversive moods. Additionally, Watts and colleagues [7] found only buprenorphine (compared to naltrexone and methadone) to be associated with reduced suicide mortality after adjusting for demographics and co-morbid medical conditions. The authors found that the reduction in suicide risk persisted through treatment periods, with a lower reduction of suicide risk early in treatment compared to later periods. Early evidence suggests that buprenorphine’s activity as a kappa opioid antagonist may be responsible for its ability to lower suicide risk quickly, as brain studies suggest that the kappa receptor plays a role in dysphoria [21]. Our study participants had only initiated buprenorphine treatment within the past month, and future studies could evaluate suicidality in individuals with OUD taking buprenorphine for a longer period of time. Alternatively, a second hypothesized explanation for buprenorphine’s efficacy in lowering suicide risk relates to the medication’s ability to foster engagement in medical care, as it is typically prescribed in an office-based, primary care setting.

Our study had several strengths. We utilized a measure of suicidality, medical and psychosocial factors, the ASI-Lite, that is well-validated in individuals with substance use disorders. Additionally, we recruited a racially and ethnically diverse sample. Our study also had several limitations. Participants were patients at FQHCs, which generally serve low-income patients, and these clinics were located in only one geographic region. Therefore, generalizability to patients in other regions, or of other socioeconomic statuses, is unknown. Additionally, participants were excluded if they demonstrated signs of active psychosis or intoxication levels that would preclude informed consent, or if they expressed immediate risk of harm to themselves or others. Therefore, rates of suicidality presented in this study may reflect conservative estimates. The rates of endorsed suicidal ideation or attempts in our sample in the past 30 days were too low to meaningfully utilize in the present analyses but could be evaluated in future studies with larger samples. Future studies may also benefit from continually assessing suicidality, and utilizing more comprehensive suicide assessment measures given that buprenorphine use is associated with reductions in suicide mortality [7]. Finally, data were collected during the COVID-19 pandemic, which may have impacted mood and suicidality in our sample, especially given that individuals with substance use disorders may have been disproportionately adversely affected by the pandemic [22].

## Conclusions

In summary, individuals with OUD report elevated rates of lifetime suicidal ideation and suicide attempts. Screening and regular monitoring for suicidality, particularly in patients with OUD of Hispanic or Latinx ethnicity, or with histories of depression, anxiety, abuse or chronic pain, is warranted. Office-based clinical settings providing MOUD would benefit from integrated behavioral health services that can improve clinical response to the mental health needs of patients.

## Data Availability

Data will be available upon study completion at https://doi.org/10.5061/dryad.stqjq2c5n.
